# Health-Risk Factors and the Prevalence of Hypertension: Cross-Sectional Findings from a National Cohort of 87 143 Thai Open University Students

**DOI:** 10.5539/gjhs.v5n4p126

**Published:** 2013-05-01

**Authors:** Prasutr Thawornchaisit, Ferdinandus de Looze, Christopher M Reid, Sam-ang Seubsman, Adrian Sleigh, Thai Cohort Study Team

**Affiliations:** 1 School of Medicine, University of Queensland, Australia; 2 School of Public Health and Preventive Medicine, Monash University, Australia; 3 School of Human Ecology, Sukhothai Thammathirat Open University, Thailand; 4 National Centre for Epidemiology and Population Health, ANU College of Medicine, Biology and Environment, The Australian National University, Australia

**Keywords:** hypertension, SES, BMI, underlying diseases, smoking, drinking, Thailand

## Abstract

**Background:**

Thailand is undergoing a health-risk transition which increases chronic diseases, particularly hypertension, as a result of a rapid transition from a developing to a developed country. This study analyzes the effect of health-risk factors such as demography, socioeconomic status (SES) and body mass index (BMI) on the prevalence of hypertension.

**Methods:**

This was a cross-sectional analysis using data obtained in 2005 from 87 143 Sukhothai Thammathirat Open University (STOU) students participating in the Thai Cohort Study (mean age 30.5 years, 54.7% female). Adjusted odds ratios of the association between risk factors and hypertension were analysed across two age groups by sex, after controlling for the confounding factors such as SES and BMI.

**Results:**

The prevalence of hypertension in men was approximately twice as high as that in women (6.9% vs 2.6%). Hypertension was associated with ageing, a lower education attainment, a higher BMI and having underlying diseases in both sexes. In men, hypertension was associated with being single, having a high income, spending more time on screens (TV & PC), cigarette smoking and drinking alcohol. In women, it was directly correlated with instant and roasted or smoked food consumption.

**Conclusions:**

Hypertension was highly associated with obesity and having underlying disease. The Thai health-risk transition is in a later stage. Thais should now be educated about the danger of high blood pressure and the protective power of a low fat and low salt diet, and a normal BMI. Cessation of smoking and moderation in alcohol intake should be promoted.

## 1. Introduction

Hypertension is a major public health challenge with its prevalence rising alarmingly worldwide (Kearney et al., 2005; Kearney, Whelton, Reynolds, Whelton, & He, 2004). It is associated with increased risk of stroke and ischemic heart disease which are major causes of global mortality, affecting low, middle and high income countries (Lawes, Vander Hoorn, & Rodgers, 2008); 62% of strokes and 49% of cardiovascular diseases are attributable to hypertension (F. J. He & MacGregor, 2007). In 2001, hypertension contributed to 7.6 million deaths (13.5% of total global mortality) and 92 million disability-adjusted life years (DALYs) (J. He & Whelton, 1997; Lawes et al., 2008).

These global hypertension trends are presenting a new challenge in middle-income countries experiencing a transition to greater longevity and chronic diseases. For example the prevalence of hypertension in Thai adults in 1991 was 5% but by 2004 it had risen to 21%, with 10 million people affected (Aekplakorn et al., 2008; Aekplakorn et al., 2012; Leelacharas, 2009). At present hypertension is still increasing in Thailand due to ageing, rising obesity and in association with rising prevalence of high blood lipids levels in the population (Banwell et al., 2009; Porapakkham, Pattaraarchachai, & Aekplakorn, 2008; Sleigh, Seubsman, & Bain, 2008). The proportion of the Thai population aged over 60 years rapidly increased from 9.5% in 2000 to 12.6% in 2010 as a result of improvements in public health (Porapakkham et al., 2008).

Urbanisation is also underway and in recent decades, rapid socio-economic growth encouraged 32% of Thais to migrate from rural to urban areas joining the 20% of Thais who had always lived in cities (L. Y. L. Lim et al., 2009). Urban Thais tend to have less healthy diets (more “junk” food) and less physical activity with a more sedentary life-style than their rural counterparts (L. Y. L. Lim et al., 2009). As a consequence, urbanisation is an important risk factor for obesity (Banwell et al., 2009). With around half of all Thais living in an urban area, 15% of the population is overweight (Asian standard body mass index (BMI) >23-24.9) and 16% is obese (BMI>25) (Banwell et al., 2009). The prevalence of overweight and obesity in educated subgroups is lower than the general population, especially for females (Banwell et al., 2009). Overall, among Thai adults, females have a higher prevalence of excess weight than males, with the most recent estimates showing 54% of females and 41% of males affected (Aekplakorn et al., 2007).

Multiple interrelated risk factors for hypertension are well documented and include ageing, obesity, diabetes mellitus, hyperlipidaemia (Messerli, Williams, & Ritz, 2007), physical inactivity (Kokkinos et al., 2006), kidney disease (Coffman & Crowley, 2008), high salt consumption (Ritz, 2010), alcohol consumption (Zilkens et al., 2005), smoking, male sex, and genetic influences (Kannel, 2009). The prevalence of hypertension increases with age in both sexes (Lloyd-Jones et al., 2009). Age is an independent predictor of future hypertension and is directly correlated with rising blood pressure (Mundal et al., 1997) from a gradual increase in peripheral vascular resistance with ageing (Franklin et al., 1997). The risk of hypertension in males and females gradually increases with increasing BMI (Meng et al., 2011). Diabetes mellitus is an important risk factor for hypertension development (Hsia et al., 2007) since it accelerates the loss of large arterial compliance leading to increased pulse and systolic pressure (Tozawa et al., 2002). There have been many reports linking sodium intake to hypertension prevalence and risk in both Western and Asian populations (Chien et al., 2008; Geleijnse, Kok, & Grobbee, 2004; Intersalt Cooperative Research Group, 1988; Sacks et al., 2001; Sun et al., 2008; Zhang, Qin, Liu, & Wang, 2010).

Thais need to understand their own risk factors for hypertension in order to put the global knowledge of hypertension into local perspective. To date, research on risk factors for hypertension among the Thai population has been restricted to specific geographical zones or limited in overall scope (Aekplakorn et al., 2012; Lwin Mm, Tassanee, Oranut, & Chaweewon, 2011; Puavilai et al., 2011; Sitthi-Amorn, Chandraprasert, Bunnag, & Plengvidhya, 1989; Vathesatogkit et al., 2012). Our large nationwide study enables examination of links among a wide array of health-risk factors and prevalent hypertension. The data are from the Thai Cohort Study, a large national cohort focused on understanding the health-risk transition in Thailand (Sleigh et al, 2008). It examines the relative importance of many risk factors associated with emerging chronic diseases in Thailand. Our hypertension analysis presented here includes assessment of socio-demography, smoking and drinking, dietary preferences, sedentary behaviour, planned and incidental exercise – elements of the overarching health-risk transition and expected to influence the occurrence of hypertension.

## 2. Methods

### 2.1 Data and Study Population

The Thai Cohort Study (TCS) includes 87 143 distance-learning students enrolled at Sukhothai Thammathirat Open University (STOU) who completed a 20-page mail-out baseline survey in 2005. Details on overall methodology have been reported elsewhere (S. Seubsman, Yiengprugsawan, Sleigh, & Thai Cohort Study, 2012; Sleigh et al., 2008). Respondents were aged from 15 to 87 years (mean age 30.5 years) and resided in all areas of Thailand. They represented well the student body at STOU, and the adult Thai population, for geographic distribution, income, age, ethnicity, religion and (modest) socio-economic status (Sleigh et al., 2008). Health-risk factors measured and analysed for their association with chronic diseases were shown to represent the health-risk transition underway in Thailand, following the eco-social model adopted for TCS (Sleigh et al., 2008).

Baseline questions focused on demography, socio-economic status (SES), work stress, personal health, history of 25 specific diseases including doctor-diagnosed hypertension, hearing, vision or dental impairment and usage of health services, social networks and personal well-being, food consumption and exercise, transport, smoking and drinking alcohol and family history.

### 2.2 Variables and Categories

Analyses included hypertension as a dependent variable. Independent variables investigated were: demography including age, sex, and urbanization status; body mass index; and lifestyle factors including food preferences and intake, physical exercise, smoking and alcohol consumption. Urbanization status was classified based on rural (R) or urban (U) residence when aged 10-12 years old and in 2005 thus producing 4 groups: lifelong ruralites (RR), urbanizers (RU), de-urbanizers (UR) and urbanites (UU). Body mass index (BMI) was calculated by the formula weight (kg)/height (m)^2^. Asian cutoffs (Banwell et al., 2009; Kanazawa et al., 2002; S. A. Seubsman et al., 2010; Weisell, 2002; Yiengprugsawan, Banwell, Seubsman, Sleigh, & Thai Cohort Study, 2012) were used for BMI classification as follows: underweight (BMI <18.5), normal (18.5 to <23), overweight (23 to <25), or obese (≥25).

Dietary assessment was restricted to food likely to be affected by the health-risk transition. We investigated frequency of specific food consumption (deep fried, instant, roast or smoked, soybean products and soft drinks) based on a five-point Likert scale ranging from less than once a month to once or more per day. Western-style fast food consumption was recorded on a three-point scale from “less than once” to “more than three times” per month. Fruit and vegetable consumption was recorded as serves eaten per day.

Physical activities - mild, moderate and strenuous exercise sessions for at least 20 minutes, or walking sessions for at least 10 minutes - were recorded as four variables each ranging from never to ≥5 times per week. House work was classified by frequency of gardening and cleaning divided into four categories ranging from ≤3 times per month to most days. For sedentary habits respondents were divided into four categories based on how many hours per day they regularly spent for sleeping, sitting and having screen time on television or computer.

Smoking status was classified into never, ex-smoker or current-smoker. Alcohol drinking status contained four categories: never, ex-drinker, occasional drinker or current-drinker.

### 2.3 Statistical Analyses

All analyses were performed using SPSS. The prevalence of hypertension and its 95% confidence interval (CI) in the cohort was calculated for each category of all variables. To minimise demographic confounding participants were separated into two groups by sex, and each group was stratified into two age groups, those ≤40 years and those aged > 40. Odds ratios (ORs) and their 95% confidence intervals were calculated for the association of hypertension with each investigated health-risk factor using bivariate logistic regression. A P-value of <5% was used as the criterion for statistical significance. Adjusted ORs were calculated using multivariable logistic regression models to control for confounding. Adjusting (confounding) variables were chosen because they demonstrated a significant association with hypertension on bivariate analysis. These variables were age, marital status, SES, BMI, physical activities, underlying diseases and personal behaviours, cigarette smoking and alcohol drinking.

## 3. Results

### 3.1 Study Population

Of the 200 000 students approached to participate in the study, 87 134 (44%) signed the consent forms and completed the questionnaires; 55% of respondents were female. The responding students represented well the social geography, socio-economic status and field of study of the Open University student body (S. Seubsman et al., 2012). Table 1 outlines the socio-demographic characteristics of study participants. The majority of participants were aged ≤ 40y with females being slightly younger than males (median 27 vs 31 years). About half of the study population resided in urban areas. The highest proportion of males and females lived in the Central and North-eastern regions respectively. Females had a moderately higher educational attainment than males while males had a higher monthly income than females. However, over half of the student population had a monthly income of less than US$250. The distribution of household assets for males and females was similar. The study population was younger, slightly more urban and had achieved a higher education attainment than the general Thai population. However, as mentioned in the Methods above, the cohort represents well the Thai population for an array of socio-demographic attributes.

**Table 1:** Socio-demographic characteristics of the 87143 participants in the Thai Cohort Study, 2005

Factor	Male		Female		Difference P-value^a^

	n	%	n	%	
**Demographic data**					
Participants	39485	45.3	47658	54.7	<0.0001
Mean age-years (SD)	32.24	(8.8)	29.07	(7.5)	<0.0001^b^
*Age group* (years)					<0.0001
≤30	19420	49.2	30831	64.7	
31-40	12993	32.9	12534	26.3	
>40	7068	17.9	4287	9.0	
*Marital status***** (married/partnered)					<0.0001
No	17941	46.8	27539	62.2	
Yes	20392	53.2	18713	37.8	
*Regions*					<0.0001
Bangkok	5715	14.6	9148	19.3	
Central	8867	22.7	12299	26.0	
North	7659	19.6	8095	17.1	
North-east	9554	24.4	8484	17.9	
East	2397	6.1	2930	6.2	
South	4933	12.6	6361	13.4	
*Urbanization status^c^*					<0.0001
Rural–rural (RR)	17607	45.3	20137	42.7	
Rural–urban (RU)	12619	32.4	14814	31.4	
Urban–rural (UR)	1699	4.4	2008	4.3	
Urban–urban (UU)	6978	17.9	10173	21.6	
**Socioeconomic status**					
*Education level*					<0.0001
High school	21750	55.1	20703	43.5	
Diploma	8903	22.6	14565	30.6	
University	8732	22.1	12253	25.8	
*Personal monthly income* (baht)^d^					<0.0001
≤7000	13609	35.4	22026	47.4	
7001–10000	8821	22.9	10977	23.6	
10001–20000	10921	28.4	9648	20.8	
>20000	5152	13.4	3803	8.2	
*Household assets*^e^ (baht)					<0.0001
Low	15911	40.5	19271	40.6	
Medium	12331	31.4	14271	30.1	
High	11024	28.1	13887	29.3	

a Chi-square test

b unpaired *t* test

c Location of residence (rural, R, or urban, U) at age 10-12 years and in 2005

d At the time of the survey in 2005, US$1 = 42 Thai baht

e Replacement value in Thai baht, divided into 3 groups: low ≤30,000, medium 30,001-60,000 and high >60,000

### 3.2 Prevalence of Factors associated with Hypertension

Overall, the prevalence of doctor-diagnosed hypertension reported by cohort members was 4.6% (6.9% of males and 2.6% of females). In both sexes, the proportion of those with hypertension increased with increasing age. Marriage or having a partner decreased the prevalence of hypertension in males, while in younger females the prevalence increased. In both younger and older males and females, a previous university degree was associated with a lower prevalence of hypertension.

The prevalence of hypertension increased with an increase of personal monthly income in all and older males. However, income had no influence on the prevalence of hypertension in females. In all groups, the prevalence of hypertension increased with an increased BMI with 11.9% of those in the obese category having a diagnosis of hypertension.

The prevalence of hypertension inversely related to the frequency of house work or gardening, especially for older males. Sedentariness was associated with increased prevalence: all males had higher prevalence of hypertension with more screen time. In contrast, sedentary activity had no association with hypertension in females.

Tables 2 and 3 reveal that the prevalence of hypertension increased in both men and women who had type 2 diabetes and high blood lipids. Hypertension was also more prevalent in all groups of participants who had reported kidney disease, except in older females. In men, ex-smokers had a higher prevalence of hypertension than non-smokers while in women, smoking had no influence on hypertension. The prevalence of hypertension increased in all and younger males who drank alcohol while it decreased in the older males. In younger females, the prevalence of hypertension increased with an increase of alcohol drinking status whereas in the older females, it decreased. In all and younger females, the prevalence of hypertension had a direct association with the frequency of instant and roasted or smoked food consumption. Older males who consumed more instant food (3-6 times/wk) had a higher prevalence of hypertension than their counterpart who consumed less (<1time/m).

**Table 2:** Prevalence of hypertension and significant risk factors in male participants

	All males			Younger males	Older males
	
	HT(n)	%Prev^a^ (95% CI)	AORs^b^(95% CI)	AORs^b^(95% CI)	AORs^b^(95% CI)
Participants	2720	6.9 (6.6-7.1)			
**Demography**					
* Age group*					
≤30 y	629	3.2 (3.0-3.5)	1		
31-40 y	872	6.7 (6.3-7.1)	1.38 (1.21-1.58)		
>40 y	1218	17.2 (16.4-18.1)	2.87 (2.47-3.33)		
P-trend			<0.0001		
* Marital status***** (married/partnered)					
No	759	4.2 (3.9-4.5)	1	1	1
Yes	1880	9.2 (8.8-9.6)	0.88 (0.78-0.99)	1.0 (0.88-1.14)	0.94 (0.72-1.23)
**Socio-economic status**					
* Education level*					
High school	1443	6.6 (6.3-7.0)	1	1	1
Diploma	585	6.6 (6.1-7.1)	1.05 (0.93-1.17)	0.96 (0.83-1.1)	1.06 (0.87-1.28)
University	682	7.8 (7.3-8.4)	0.87 (0.78-0.98)	0.82 (0.7-0.96)	0.9 (0.76-1.07)
P-trend			<0.032	<0.016	0.313
* Personal monthly income* (baht)^c^					
≤7000	541	4.0 (3.7-4.3)	1	1	1
7001-10000	470	5.3 (4.9-5.8)	1.18 (1.02-1.36)	1.17 (0.99-1.37)	1.39 (0.98-1.96)
10001-20000	912	8.4 (7.8-8.9)	1.13 (0.98-1.30)	1.26 (1.06-1.49)	1.3 (0.98-1.73)
>20000	737	14.3 (13.4-15.3)	1.34 (1.13-1.59)	1.16 (0.91-1.47)	1.7 (1.3-2.35)
P-trend			<0.002	0.075	<0.0001
** BMI classification^d^**					
Underweight	47	2.0 (1.42-2.6)	0.62 (0.45-0.86)	0.61 (0.42-0.87)	0.56 (0.26-1.24)
(BMI <18.5)					
Normal	739	3.8 (3.6-4.1)	1	1	1
(18.5≤BMI<23)					
Overweight	623	7.4 (6.8-8.0)	1.42 (1.26-1.61)	1.5 (1.28-1.75)	1.38 (1.13-1.7)
(23 ≤ BMI <25)					
Obese	1242	14.0 (13.3-14.8)	2.41 (2.16-2.69)	2.66 (2.31-3.06)	2.23 (1.85-2.67)
(BMI ≥25)					
P-trend			<0.0001	<0.0001	<0.0001
**Sedentary habits**					
* House work/gardening*					
Most days	900	6.6 (6.2-7.0)	1	1	1
3-4 times/wk	362	6.8 (6.1-7.5)	1.13 (0.98-1.31)	1.1 (0.92-1.31)	1.16 (0.92-1.47)
1-2 times/wk	734	6.8 (6.4-7.3)	1.16 (1.03-1.30)	1.02 (0.88-1.19)	1.37 (1.13-1.66)
<3 times/m	683	7.5 (7.0-8.1)	1.09 (0.96-1.23)	0.97 (0.83-1.14)	1.26 (1.04-1.53)
P-trend			0.14	0.743	<0.008
* TV/PC watching* (hours/day)					
≤1 hr	612	6.9 (6.4-7.5)	1	1	1
2 hr	820	7.1 (6.6-7.6)	0.96 (0.8-1.15)	1.11 (0.94-1.32)	0.96 (0.8-1.15)
3 hr	544	6.7 (6.2-7.3)	1.18 (0.95-1.46)	1.11 (0.93-1.34)	1.18 (0.95-1.46)
≥4 hr	695	6.8 (6.3-7.3)	1.39 (1.13-1.71)	1.2 (1.0-1.42)	1.39 (1.13-1.71)
P-trend			<0.001	0.066	<0.001
**Underlying diseases**					
*Diabetes mellitus type 2*					
No	2500	6.4 (6.2-6.7)	1	1	1
Yes	220	41.7 (37.5-46.0)	3.63 (2.94-4.49)	4.37 (3.0-6.36)	3.19 (2.47-4.12)
*High lipids*					
No	1748	5.0 (4.8-5.3)	1	1	1
Yes	972	20.8 (19.6-21.9)	2.38 (2.14-2.65)	3.15 (2.72-3.66)	1.85 (1.6-2.15)
*Kidney diseases*					
No	2557	6.6 (6.4-6.9)	1	1	1
Yes	163	16.3 (14.0-18.6)	2.16 (1.75-2.66)	2.64 (2.02-3.45)	1.55 (1.11-2.15)
**Personal behaviours**					
*Smoking status*					
Never	990	5.6 (5.3-6.0)	1	1	1
Ex-smoker	1152	8.9 (8.4-9.4)	1.21 (1.09-1.34)	1.21 (1.06-1.39)	1.25 (1.06-1.48)
Cur-smoker^e^	525	6.5 (6.0-7.1)	1.04 (0.92-1.18)	1.0 (0.85-1.17)	1.14 (0.92-1.41)
P-trend			0.283	0.952	0.741
*Drinking status*					
Never	224	5.4 (4.7-6.1)	1	1	1
Ex-drinker	393	8.9 (8.0-9.7)	1.26 (1.03-1.54)	1.45 (1.1-1.91)	1.0 (0.74-1.35)
Occ – drinker^f^	1681	6.3 (6.0-6.6)	0.99 (0.84-1.17)	1.25 (1.0-1.57)	0.67 (0.51-0.88)
Reg – drinker^g^	391	10.1 (9.2-11.1)	1.33 (1.08-1.63)	1.58 (1.21-2.08)	1.02 (0.74-1.41)
P-trend			0.084	0.068	0.232
**Food consumption habit**					
* Instant food*					
<1 time/m	713	9.1 (8.5-9.7)	1	1	1
1-3 times/m	1018	7.0 (6.6-7.4)	0.99 (0.88-1.11)	1.09 (0.92-1.29)	0.88 (0.75-1.04)
1-2 times/wk	604	6.0 (5.5-6.4)	1.06 (0.93-1.21)	1.08 (0.9-1.29)	1.04 (0.85-1.29)
3-6 times/wk	310	5.5 (4.9-6.1)	1.14 (0.96-1.34)	1.02 (0.83-1.26)	1.65 (1.23-2.21)
≥1 times/d	51	4.8 (3.5-6.1)	1.04 (0.73-1.47)	1.2 (0.83-1.75)	0.36 (0.12-1.06)
P-trend			0.161	0.706	0.188

a Prevalence of Hypertension

b Adjusted Odd ratios were from logistic regression models of hypertension adjusted for age, marital status, socioeconomic status, BMI classification, physical activities, underlying diseases, and personal behaviours

c In 2005 US$1 = 42 Thai baht

d Asian standard BMI classification

e Current-smoker

f Occasional drinker

g Regular drinker

**Table 3:** Prevalence of hypertension and significant risk factors in female participants

	All females			Younger females	Older females
	
	HT(n)	%Prev^a^ (95%CI)	AORs^b^(95% CI)	AORs^b^(95% CI)	AORs^b^(95% CI)
Participants	1250	2.6	(2.48-2.77)		
**Demography**					
*Age group*					
≤30 y	532	1.7 (1.6-1.9)	1		
31-40 y	359	2.9 (2.6-3.2)	1.52 (1.29-1.8)		
>40 y	359	8.4 (7.5-9.2)	3.77 (3.07-4.61)		
P-trend	<0.0001			
*Marital status***** (married/partnered)					
No	564	2.1 (1.9-2.2)	1	1	1
Yes	650	3.5 (3.2-3.7)	1 (0.88-1.16)	1.19 (1.02-1.38)	0.94 (0.71-1.26)
*Education level*					
High school	579	2.8 (2.6-3.0)	1	1	1
Diploma	389	2.7 (2.4-2.9)	1.02 (0.88-1.18)	0.95 (0.80-1.12)	1.06(0.78-1.44)
University	277	2.3 (2.0-2.5)	0.7 (0.59-0.83)	0.66 (0.53-0.81)	0.8 (0.59-1.1)
P-trend			<0.0001	<0.0001	0.248
**BMI classification^c^**
Underweight (BMI<18.5)	142	1.4 (1.2-1.6)	0.83 (0.67-1.02)	0.8 (0.45-1.01)	0.57 (0.23-1.44)
Normal (18.5≤BMI<23)	556	2.0 (1.8-2.2)	1	1	1
Overweight(23≤BMI<25)	162	3.6 (3.0-4.1)	1.31 (1.07-1.6)	1.46 (0.97-2.24)	1.14 (0.8-1.64)
Obese (BMI≥25)	376	8.0 (7.2-8.8)	3.1 (2.66-3.61)	3.05 (2.05-3.99)	3.3 (2.48-4.38)
P-trend			<0.0001	<0.0001	<0.0001
**Underlying diseases**					
*Diabetes mellitus type 2*					
No	1194	2.5 (2.4-2.7)	1	1	1
Yes	56	26.9 (20.9-33.0)	5.68 (3.94-8.21)	7.02 (4.28-11.51)	4.19 (2.42-7.24)
*High lipids*					
No	990	2.2 (2.1-2.4)	1	1	1
Yes	260	8.9 (7.9-10.0)	2.4 (2.02-2.86)	2.77 (2.2-3.48)	2.08 (1.6-2.7)
*Kidney diseases*					
No	1155	2.5 (2.4-2.6)	1	1	1
Yes	95	7.4 (6.0-8.8)	3.0 (2.35-3.83)	3.36 (2.59-4.36)	1.41 (0.71-2.81)
**Personal behaviours**					
*Drinking status*					
Never	481	2.6 (2.4-2.8)	1	1	1
Ex-drinker	107	3.2 (2.6-3.9)	1.14 (0.89-1.45)	1.28 (0.97-1.69)	0.84 (0.49-1.42)
Occ-drinker^d^	633	2.6 (2.4-2.8)	1.08 (0.94-1.23)	1.25 (1.06-1.46)	0.72 (0.55-0.94)
Reg-drinker^e^	12	3.9 (1.7-6.1)	1.46 (0.79-2.72)	1.81 (0.89-3.67)	0.92 (0.26-3.19)
P-trend			0.238	<0.009	<0.021
**Food consumption habits**					
*Instant food*					
<1 time/m	280	3.3 (3.0-3.7)	1	1	1
1-3 times/m	475	2.5 (2.3-2.8)	1.05 (0.89-1.25)	1.03 (0.83-1.29)	1.11 (0.84-1.48)
1-2 times/wk	284	2.3 (2.1-2.6)	1.11 (0.91-1.35)	1.06 (0.84-1.34)	1.11 (0.76-1.64)
3-6 times/wk	153	2.3 (2.0-2.7)	1.14 (0.9-1.44)	1.05 (0.8-1.37)	1.16 (0.65-2.08)
≥1 times/d	52	3.7 (2.7-4.7)	1.97 (1.4-2.79)	1.89 (1.31-2.73)	0.59 (0.07-5.08)
P-trend			<0.012	<0.043	0.66
*Roasted/smoked food*					
<1 time/m	229	3.0 (2.6-3.4)	1	1	1
1-3 times/m	427	2.5 (2.3-2.7)	1.03 (0.87-1.21)	1.0 (0.78-1.27)	0.93 (0.69-1.26)
1-2 times/wk	356	2.5 (2.2-2.7)	1.09 (0.91-1.31)	1.15 (0.9-1.47)	1.23 (0.86-1.75)
3-6 times/wk	187	2.7 (2.3-3.1)	1.14 (0.93-1.4)	1.29 (0.99-1.69)	0.91 (0.54-1.54)
≥1 times/d	38	3.1 (2.1-4.0)	1.39 (1.08-1.77)	1.57 (1.04-2.38)	0.49 (0.11-2.17)
P-trend			<0.013	<0.004	0.906

a Prevalence of Hypertension

b Adjusted Odd ratios were calculated from logistic regression models of hypertension adjusted for age, marital status, socioeconomic status, BMI classification, physical activities, underlying diseases, and personal behaviours

c Asian standard BMI classification

d Occasional drinker

e Regular drinker

### 3.3 Factors not associated with Hypertension (Data not Shown)

In both males and females, factors that were not independently and statistically significantly related to the prevalence of hypertension were urbanization status, household assets, sleeping and sitting time, physical activities, and food consumption habits including deep fried food, soft drink, Western fast food, fruit, vegetables and soy products.

In males, roasted or smoked foods consumption had no effect on hypertension. In females, the factors that had no association with hypertension were personal monthly income, housework or gardening, television and computer watching time and cigarette smoking.

## 4. Discussion

The TCS has been the first and largest nationwide study of a wide array of locally important risk factors for hypertension in Thailand. Hypertension was shown to be strongly associated with age, sex, obesity and underlying diseases including type 2 diabetes, high blood lipids and kidney disease. The observation that the risk of hypertension increases with age for both men and women is consistent with other findings (Jo et al., 2001; Meng et al., 2011; Rampal, Rampal, Azhar, & Rahman, 2008). Increased blood pressure with ageing reflects reduction of large arterial compliance, causing increased arterial stiffness (Tozawa et al., 2002). This process affects ageing Thais.

The prevalence of hypertension in males was more than twice that in females. Whilst this observation is comparable with reports from other countries (Bhalla, Fong, Chew, & Satku, 2006; Choi et al., 2006; Rampal et al., 2008; Su et al., 2008), the actual prevalence rates in the TCS were much lower than that of the Third National Thai Health Examination Survey (Aekplakorn et al., 2008) in 2004 (male: 6.9% vs 23.3%; female: 2.6 vs 20.9%). Some of this discrepancy could be a result of a (double) healthy cohort effect (Last, 2001) – the cohort constituting the STOU student body and the cohort of STOU students who joined our study would be expected to include relatively healthy individuals. As well, the low rate of hypertension in the cohort must in part be due to the majority of participants in the current study being younger than in the general adult population. Data from the National Health and Nutrition Examination Survey (NHANES) recorded that prevalence in males was higher than that in females until 45 years of age (Lloyd-Jones et al., 2009). In Thailand, hypertension awareness, treatment and control is higher in females than in males (Aekplakorn et al., 2012).

We found the risk of hypertension was linearly associated with BMI in all age groups of males and females which was compatible with previous studies in Thailand (Aekplakorn et al., 2008) and in other countries (Bassett, Fitzhugh, Crespo, King, & McLaughlin, 2002; Joshi, Lim, & Nandkumar, 2007; Pang et al., 2010; Tesfaye et al., 2007). The association has been shown in other studies to follow a dose-response pattern (Jo et al., 2001) with each unit of BMI increasing the risk of developing hypertension by 9% (Pang et al., 2010). Obesity is a major risk factor for hypertension (Pang et al., 2010) and a number of potential mechanisms through which this impacts on blood pressure have been identified. These mechanisms include endothelial dysfunction, abnormal renal function, enhanced sympathetic nervous system (SNS) and renin-angiotensin-aldosterone system (RAAS) activity leading to vasoconstriction and intravascular sodium and water retention (Rahmouni, Correia, Haynes, & Mark, 2005). In Thailand, obesity is becoming a critical public health challenge with prevalence high and rising (Aekplakorn et al., 2007). In 2004, overweight or obesity affected about one in five or one in 10 Thai adults, respectively (Aekplakorn et al., 2007).

As with previous studies (Al Ghatrif et al., 2011; Fukui et al., 2011; Levin et al., 2010); all participants with type 2 diabetes had a higher risk of hypertension than those without diabetes. The risk increased with an increase of fasting plasma glucose level (Aekplakorn et al., 2008). Diabetes is a predictor of hypertension (Joshi et al., 2007) since it facilitates diabetic nephropathy (Al Ghatrif et al., 2011) and damages the arterial wall, reducing elasticity (Levin et al., 2010). As well, hyperinsulinemia stimulates the SNS and RAAS (Levin et al., 2010).

In all age groups, high blood lipid levels were strongly associated with increased prevalence of hypertension as has been previously reported (Fukui et al., 2011; Halperin et al., 2006; Laaksonen et al., 2008; Sesso, Buring, Chown, Ridker, & Gaziano, 2005). A study of association between plasma lipid levels and hypertension in women (Sesso et al., 2005) showed that the risk of elevated blood pressure rose with an increase of total cholesterol (TC) and low-density lipoprotein cholesterol (LDL-C) level and fell with an increase of high-density lipoprotein cholesterol (HDL-C) level. In men, hypertension risk increased with elevated triglyceride (TG) and LDL-C and fell with high HDL-C (Laaksonen et al., 2008). High blood lipids impair endothelial function reducing nitric oxide production leading to arteriosclerosis (Halperin et al., 2006; Laaksonen et al., 2008; Sesso et al., 2005). Elevated blood lipids also associate with SNS overactivity (Halperin et al., 2006) and RAAS boosts angiotensin (Laaksonen et al., 2008).

Cohort men and women with kidney disease had a significantly higher risk of hypertension than those without kidney disease with the exception of older females. This observation may be due to the small number of older females in the current study. Other studies have shown that the risk of hypertension directly associates with the serum creatinine level (Johnston & Davison, 1993) and inversely associates with the glomerular filtration rate (Buckalew et al., 1996), both reflecting the severity of kidney dysfunction. Overactivity of renin-angiotensin due to nephropathy may enhance reabsorption of sodium at the proximal tubule (Johnston & Davison, 1993).

Married males had a significantly lower risk of hypertension than single males which was consistent with a similar finding in Polish males (Lipowicz & Lopuszanska, 2005). Single, separated and widowed males are significantly more likely to smoke cigarettes than married males (Molloy, Stamatakis, Randall, & Hamer, 2009). In addition, they have significantly higher psychological distress (Molloy et al., 2009) while married men have higher personal wellbeing indices, which include personal health and future security (Yiengprugsawan, Seubsman, Khamman, Lim, & Sleigh, 2010). In contrast, married younger females had a significantly higher risk of hypertension than their single counterparts. This may be because married females in the present study tended to be more overweight and obese than single females.

Higher income TCS males were older, had higher rates of current smoking, were more likely to be overweight and obese, and consumed more alcohol and unhealthy food. The pattern in TCS males was similar to that in developing countries which reported that hypertension increased with an increase of family income (Meng et al., 2011). In TCS females, income had no detected influence on hypertension. Cohort females may be more aware of their health and well-being and they tended to maintain normal weight. Culturally Thai females are protected against alcohol and cigarettes as both are considered unsuitable for females. They are also more likely to regularly visit a doctor, especially now it is free of charge under universal health coverage (Yiengprugsawan, Seubsman, Lim, Sleigh, & Thai Cohort Study, 2009). This pattern resembles that in developed countries (Bassett et al., 2002; Bell, Thorpe, & Laveist, 2010; Jo et al., 2001; Kaplan, Huguet, Feeny, & McFarland, 2010).

A higher level of educational attainment was associated with a lower risk of hypertension in both sexes, especially in the younger groups, similar to the pattern in developed countries (Choi et al., 2006; Halanych et al., 2010; Rampal et al., 2008; Schwandt, Coresh, & Hindin, 2010). People with higher education had better health and knowledge (Min, Chang, & Balkrishnan, 2010). Household assets had no influence on hypertension in both sexes perhaps because they depend on many factors such as individual income and educational attainment. Higher income was likely associated with increased risk whereas higher educational achievement was related to decreased risk.

In older males, a higher frequency of house work or gardening was associated with decreased risk of hypertension. It was consistent with a previous study which reported that in adults yard work and gardening were significantly associated with a decrease of blood pressure (Thurston, Sherwood, Matthews, & Blumenthal, 2011). Spending more time on TV or PC was associated with an increased risk of hypertension in males, especially older men, as noted in other studies (Aadahl, Kjaer, & Jorgensen, 2007; Wells et al., 2008). It may be a result of an increase of BMI (Wells et al., 2008). Spending more time on TV or PC implies less physical activity and increasing BMI (Aadahl et al., 2007). More alcohol, unhealthy food such as snacks and sweets and less vegetables and fruits have also been reported (Hu et al., 2001).

In all, younger and older males, ex-smokers had a significantly higher risk of hypertension than non-smokers which was compatible with a study of hypertension and associated factors in China (Pang et al., 2010). However cigarette smoking had no impact on hypertension in current smoking males; this was likely due to a small number of smokers in this study. Similarly, it had no effect on females because only a very small number of females smoked. A study in males (Niskanen et al., 2004) reported that increased risk of hypertension was directly associated with an increase of daily cigarette smoking in a dose-response manner. Cigarette smoking may cause renal impairment (Niskanen et al., 2004) and atherosclerosis from vascular endothelial damage which leads to a reduction of nitric oxide production (N. Toda & H. Toda, 2010). In this study, smoking was a risk factor for hypertension in males.

In younger males and females, drinking alcohol increased risk of hypertension while in older males, risk decreased. Young people tended to drink heavily more often, older people drank less. The results were similar to other studies (Jo et al., 2001; Murray et al., 2002; Pang et al., 2010; Thadhani et al., 2002). Half a standard drink of alcohol consumed daily significantly decreased hypertension risk but more than 2 drinks increased risk (Halanych et al., 2010; Thadhani et al., 2002). The link between hypertension and daily alcohol consumption was recorded as a J-shaped curve and all types of beverages such as beer, wine and liquor had a similar effect (Thadhani et al., 2002).

Most fruit, vegetables and foods had no direct influence on hypertension. However, consumption of instant food, generally containing high levels of fat, carbohydrate and salt, while low in fibre, vitamins and minerals, was associated with an increased risk of hypertension in older males and in females, especially younger females. Roasted or smoked foods, containing proteins and fat, were associated with an increased risk in females, particularly younger women. Thais usually have food with added salty seasoning (soy or fish sauces) and sodium consumption has a strong and direct association with hypertension (Jolly et al., 2011; Sacks et al., 2001; Stamler, Caggiula, & Grandits, 1997; Zhang et al., 2010). In contrast, consumption of carbohydrate (Brehm, Seeley, Daniels, & D’Alessio, 2003; Jolly et al., 2011; Ludwig et al., 1999) and proteins from meats and vegetables (Alonso, Beunza, Bes-Rastrollo, Pajares, & Martinez-Gonzalez, 2006; Elliott et al., 2006; Sadakane et al., 2008; Umesawa et al., 2009; Wang et al., 2008) had no effect on hypertension.

The information we present here comes from the largest cohort study in Thailand and we could evaluate many risk factors and their effects on hypertension. Even though participants are younger, more urban and more educated, they represent the Thai population very well for geography and many socio-demographic and socio-economic factors (S. Seubsman et al., 2012). The results from this study will reflect the future trend of hypertension since participants, educationally and aspirationally, represent the coming Thai generation. Cohort participants are well educated and well acquainted with mail-based questionnaires; so the reliability of self-report in our study is likely to be higher than that for the general population.

As it is a cross-sectional report and each variable is only measured at one point in time, it is not possible to prove the association between causes and effects. Prevalence of self-reported hypertension may be higher in participants having co-morbidity (including diabetes, high blood lipids or kidney disease) than those without since they tend to visit physicians regularly for their disease follow-up. Therefore, their hypertension is more likely to be identified by a doctor than their normal counterparts. However, it should be noted that blood pressure measuring stations have been widely used in the Thai community over the last 5 years and virtually all Thais have been exposed to free blood pressure measurement. Validity of self-reported weight and height in this cohort showed that participants were over-reporting height and under-reporting weight, but not causing a large error (Lim, Seubsman, & Sleigh, 2009). The sensitivity and specificity of self-report for obesity (BMI ≥25 kg/m^2^) were 74.2% and 97.3%, respectively, for men and 71.9% and 100% for women so overall, self-report provided economical and valid measures of BMI (L. L. Lim et al., 2009). In addition, a validity study (n=480) of self-reported hypertension in our cohort demonstrated that overall accuracy was 75%; sensitivity was high (82%) and that negative reports were usually accurate (86%) (Prasutr Thawornchaisit, 2013, under review). Among those who are well educated, we found self-report of hypertension is an economical and reasonably accurate method of screening populations for hypertension.

In conclusion, our study has shown that the factors associated with hypertension in Thailand are similar to those reported elsewhere. The prevalence of hypertension in males was more than twice as high as in females. Hypertension was strongly associated with ageing, a higher BMI and having underlying diseases. It was also moderately associated with a lower educational attainment in both sexes. It is an important emerging health problem since Thai people are living longer and the obesity rate is rising. Public health policy should build on the public blood pressure measuring stations and move on to more formal health education focused on education, physical activity and healthy food consumption. In order to decrease mortality from stroke and heart disease in Thais, a national health strategy should be developed to detect, treat and prevent hypertension.
